# From Offline to Online: Understanding Chinese Single Mothers’ Uncertainty Management in Interpersonal and Online Contexts

**DOI:** 10.3389/fpsyg.2022.845760

**Published:** 2022-05-06

**Authors:** Kai Kuang, Xiaoman Zhao, Iccha Basnyat, Tianping He

**Affiliations:** ^1^School of Journalism and Communication, Tsinghua University, Beijing, China; ^2^School of Journalism and Communication, Renmin University of China, Beijing, China; ^3^Global Affairs Program, Department of Communication, George Mason University, Fairfax, VA, United States; ^4^School of Sociology and Population Studies, Renmin University of China, Beijing, China

**Keywords:** Chinese single mothers, theory of motivated information management, cultural norm, perceived stigma, information management, support seeking

## Abstract

Divorced and unwed single motherhood is heavily stigmatized in Chinese cultural context, preventing Chinese single mothers from actively seeking the information and support needed and negatively impacting their wellbeing. Drawing on the theory of motivated information management (TMIM), this study tested how perceived stigma and cultural norms influenced Chinese single mothers’ search for information and social support from families, friends as well as from online communities. Using two-wave data collected from 226 single mothers, findings support the utility of the TMIM in explaining information management and support seeking behaviors and contribute to situating the TMIM process within larger socio-cultural contexts. Practical implications regarding how to facilitate more effective uncertainty management and enhance Chinese single mothers’ wellbeing in interpersonal vs. online contexts are discussed.

## Introduction

Recent social changes in China have led to significant increase in divorce and out-of-wedlock childbirth, creating a large group of single mothers with dependent children (Wang and Zhou, 2010; Lu and Xie, 2013). According to Ministry of Civil Affairs of the People’s Republic of China (2020), about 4.7 million marriages ended in 2019, a 5.4% increase compared to the previous year. In metropolitan cities such as Beijing and Shanghai, the divorce rate is estimated to be as high as 40% of those married (Fu and Wang, 2019). Regarding unwed single mothers, no official statistics exist because non-marital childbearing remains unrecognized legally in China. However, local predictions suggest a growing number of unwed single mothers despite vigorous control over out-of-wedlock childbirth (Zhou and Wang, 2013). The 2010 National Census data estimated that 90 million people did not have household registration and a large portion were illegitimate children, accounting for about 7.1% of the country’s total population (Sun, 2018). Due to deep-seated cultural norms against single motherhood, Chinese single mothers commonly experience stigma and stress that threaten their physical and psychological wellbeing (Zhao and Basnyat, 2021).

Previous research on single motherhood suggests that single mothers draw on personal networks, including family ties and friends, for information and support (Ranci, 2010; Lumino et al., 2016). However, in Chinese cultural contexts, stigmatization associated with single motherhood is much stronger compared to that in Western countries (Zhao and Basnyat, 2021). Importantly, traditional familial values are still dominant in China, preserving marriage as fulfilling gender expectations and disapproving divorce as a socio-cultural taboo and a disgrace to the family (Wang and Zhou, 2010; Fu and Wang, 2019). Unwed motherhood remains a gray area legally, as neither the Population and Family Planning Law nor the Marriage Law contains language explicitly allowing or forbidding single parenthood. In many places, single mothers still face fines or other forms of penalties for giving birth outside of marriage (Wang, 2021).^1^ Due its lack of legal legitimacy and non-compliance with the National Population Control Policy (Cao, 2015), single motherhood is commonly considered a shame for the mother herself as well as her family. Overall, single motherhood in China is associated with uncertainty, ambivalence, and precarity, all of which pose major challenges to women’s mental and physical health (Beck et al., 2010; Meier et al., 2016). These uncertainties may drive Chinese single mothers’ communication behaviors. Meanwhile, perceptions of stigma and social norms in the unique Chinese cultural context may prevent single mothers from accessing or actively seeking the information and support needed (Hung et al., 2004). In this study, we draw from the theory of motivated information management (TMIM; Afifi and Weiner, 2004) to investigate the mechanism of Chinese single mothers’ information management and support seeking processes.

## Contextualizing Single Motherhood in China

Being a single mother, in any cultural context, is challenging. Empirical findings have related single motherhood with great loss and pain to the individual, including negative emotions (e.g., anger, hurt, blame, shame), reappraisal of self-esteem as well as social isolation (Hung et al., 2004). Physical and mental health symptoms such as insomnia and stress have been reported amidst the marital turmoil and the decision over a single motherhood (Liang et al., 2019). In addition, as a departure from the “ideal” family, single motherhood also poses a “risk” to the mother’s own identity of a “good” mother, as women are considered to have condemned their child to a fatherless life (Graham, 2018). Uncertainties also arise in how to manage the continuity of the relationship with ex-spouse for co-parenting after losing the former attachment figure (Coleman and Glenn, 2009; Chen et al., 2021), leading to more anxiety for being “not good enough” parents (Graham, 2018). As a result, the experience of single motherhood is characterized by ambivalence and uncertainty, because single mothers need to address challenges of economic vulnerability for solo childrearing, alimony and child support, as well as both identity- and relationship-related challenges (Hung et al., 2004; Hertog, 2009).

In a social and cultural context that legitimates an overwhelmingly negative connotation of divorced or unwed single motherhood, Chinese single mothers encounter even more difficulties and uncertainties. The rapid social and economic changes in China within recent decades are expected to lead to increasing breakdowns in marital relationships (Anderson et al., 2012). Juxtaposed to this is the traditional cultural emphasis on familism, which still lingers in Chinese society and disapproves divorced and out-of-wedlock childbirth (Hung et al., 2004). Deep-seated Confucian family values, with the moral logics of self-discipline for social and family order, still prevail in China (Jeffreys, 2006). Marriage is deemed a solemn and important event for every individual and is expected to last for a lifetime (Higgins et al., 2002). In Chinese contexts where the social harmony rhetoric is deeply entrenched, divorced and unwed childbirth rank among the highest of cultural taboos (Fu and Wang, 2019). Divorce is discouraged and seen as a highly undesirable social and moral option, even when spouses experience severe difficulties in marriage (Fu and Wang, 2019). Even stronger stigmatization exists surrounding unwed motherhood, as premarital birth in particular is considered to bring shame on not only the individual but also her family (Zhang et al., 2014).

As a result, Chinese single mothers need information and support to mobilize resources from their personal and community networks (Hung et al., 2004; Liang et al., 2019), but the social stigma attached to single motherhood may prevent them from seeking such information or support (Hung et al., 2004). In general, individuals avoid direct communication that could cause others to lose face, which may inhibit disclosure about single motherhood (Epstein et al., 2012). For these reasons, Chinese single mothers’ information and support seeking can be a complicated communication process. However, there is little empirical research on factors that would account for Chinese single mothers’ communication behaviors in response to the uncertainties and challenges they face.

Family and friends can offer useful advice and emotional support to single mothers. Online communities also serve as valuable resources for socially stigmatized individuals to access information and support safely (Niezen, 2013). Unlike offline support groups, online support groups enable anonymity and thereby reduce the risks typically associated with offline information seeking (Gavin et al., 2008). Existing research in health communication supports the association between computer-mediated information and support seeking and recipients’ empowerment and health improvement (Van Uden-Kraan et al., 2009; Oh and Lee, 2012). Especially for marginalized and stigmatized groups, online information and support can be important alternatives (Chung, 2013; Smedley et al., 2015). Therefore, we examine Chinese single mothers’ information seeking and support seeking behaviors from sources including family members, friends, as well as online communities. The theory of motivated information management (TMIM; Afifi and Weiner, 2004) serves as a useful theoretical framework to explain the process through which Chinese single mothers seek information and support.

## Theory of Motivated Information Management

The TMIM (Afifi and Weiner, 2004) explains individuals’ decisions to seek or avoid information about personally significant issues. The theory has been used to account for information management regarding challenging, taboo, or sensitive matters such as sexual health (Dillow and Labelle, 2014), end-of-life preferences (Rafferty et al., 2015), and family health history (Hovick, 2014).

The TMIM posits that individuals go through a three-phase process before making a decision to seek information, avoid information, or cognitively reassess their states and needs for information (Afifi and Weiner, 2004). The first phase—*interpretation*—involves the recognition of an uncertainty discrepancy on an important issue, as for individuals to initiate the information management process, the issue has to be one that is of significance to the self, an important other, or a particular close relationship. Uncertainty refers to a cognitive state that occurs when details of a situation are ambiguous, complex, unpredictable, or probabilistic (Brashers, 2001), which is typical of single motherhood especially in China. Uncertainty discrepancy refers to the difference between one’s actual and desired levels of uncertainty (Afifi and Weiner, 2004). For instance, an individual with a good deal of knowledge about an issue may still experience uncertain discrepancy if they desire a higher level of certainty. The theory proposes that uncertainty discrepancy drives individuals’ information management decisions. Specifically, becoming aware of a higher- or lower-than-desired uncertainty level is associated with a range of emotions, including but not limited to anxiety (Afifi and Morse, 2009). Given the stigmatized single motherhood context of this study, we focus on negative emotions and propose:

**H1:** Uncertainty discrepancy about single motherhood is positively associated with negative emotions.

To cope with the negative emotions associated with uncertainty discrepancy, individuals may engage in different information management strategies. The decision to seek or avoid information is mediated by individuals’ assessments of outcome expectancies and efficacy in the second phrase—*evaluation* (Afifi and Weiner, 2006). Outcome expectancies involve perceptions of the possible rewards and costs associated with the information management strategy (e.g., the outcomes of seeking information). Some might consider information seeking to be time-consuming, difficult, and associated with potential identity or relationship threat, whereas others may perceive it to be simple, straightforward, and safe (e.g., Fowler and Afifi, 2011). In the context of Chinese single motherhood, seeking information from family, friends, and online resources may be seen as negative, or that the process of seeking information may have undesirable relational implications due to stigma.

Outcome expectancies influence one’s efficacy assessments. Efficacy involves individuals’ perceptions of their ability to successfully perform a behavior or produce an outcome. The TMIM specifies three types of efficacy, which, in conjunction with outcome expectancies, may predict individuals’ selection and pursuit of a specific information management strategy. Communication efficacy refers to an individual’s belief about whether he or she has the skills to raise questions and seek information from others. It reflects a person’s belief in their ability to successfully enact a particular information-seeking strategy. Coping efficacy describes an individual’s belief in their ability to pragmatically and emotionally cope with the information acquired (e.g., whether a Chinese single mother can handle the anticipated discovery that their family has a highly negative opinion about single mothers). Target efficacy involves the assessment of information providers in terms of their ability to provide information about the target issue (e.g., whether they have access to the information) and their perceived honesty with the information provided (e.g., whether they will be completely honest and forthcoming despite the sensitivity of the issue). According to the TMIM, these efficacy assessments directly impact individuals’ information-management choices and mediate the effect of outcome expectancies on that choice (Afifi and Weiner, 2004).

Finally, in the *decision* phase, individuals decide whether to seek information, avoid information, or engage in cognitive reappraisal based on their outcome expectancies and efficacy judgments (Afifi and Weiner, 2004). Information seeking involves asking questions or initiating conversations. Information avoidance occurs when individuals take deliberate steps to prevent exposure to specific knowledge, likely as a result of negative outcome expectancies and low efficacy assessments. Drawing on the TMIM, we propose the following hypotheses:

**H2:** Uncertainty discrepancy-related negative emotions is negatively associated with (a) outcome expectancies and (b) efficacy assessments.

**H3:** Outcome expectancies are positively associated with efficacy assessments.

**H4:** Efficacy assessments are (a) positively associated with information seeking and (b) negatively associated with avoidance.

## Support Seeking Behaviors

Seeking and receiving social support from trusted ones can contribute substantially to both mental and physical health (Mortenson, 2009). Meanwhile, seeking social support from friends and family involves risks and relational consequences that may inhibit support seeking behaviors. These risks and concerns include but are not limited to worries of burdening others, feelings of shame, and fear of a loss of face (Epstein et al., 2012). Cultural norms in Chinese cultures also discourage people from expressing emotional distress to others for fear of disturbing relational harmony (Taylor et al., 2004). In view of the delicacy and complexity of the support process, this study also examines Chinese single mothers’ support seeking behaviors in response to uncertainty discrepancies and associated emotions.

According to Barbee and Cunningham’s (1995) Sensitive Interactions Systems Theory (SIST), support seeking behaviors can be direct or indirect. Direct support seeking can be verbal, which involve a factual expression of need for help or non-verbal indicators of emotional distress, such as crying or dramatic pouting. Indirect support seeking, by contrast, involves more subtle and less informative strategies, such as implicit complains about a situation or subtle displays of negative affect in the form of sighing, sulking, or fidgeting. Support outcome expectations are found to influence support seekers’ decisions about whether and how they seek support (Barbee and Cunningham, 1995). In addition, higher efficacy assessments may motivate more direct support seeking, whereas lower efficacy levels may lead to indirect support seeking strategies to maintain face or to avoid obligating the potential supporters through a direct approach (Barbee and Cunningham, 1995; Kuang and Wang, 2022). Drawing on the SIST, we propose:

**H5:** Efficacy assessments are (a) positively associated with direct support seeking and (b) negatively associated with indirect support seeking.

## The Roles of Cultural Norm and Perceived Stigma

Although the TMIM is highly useful in explaining and predicting individuals’ information management process, the influence of cultural factors on this process has not been examined in the literature. In this study, we investigate how perceptions of cultural norms against single motherhood and perceived stigma may influence Chinese single mothers’ communication with others offline and online.

### Subjective Cultural Norms

Cultural norm may impact information seeking motivations in the context of single motherhood (Epstein et al., 2012; Fu and Wang, 2019). The theory of planned behavior (Fishbein, 1975; Azjen and Fishbein, 1980) conceptualizes subjective norms as individuals’ beliefs about the extent to which other people who are important to them, including best friend, parents, and significant others, think they should or should not perform particular behaviors. In Chinese cultural contexts, the shared social expectation is that women should not bear a child out of wedlock (i.e., women should not be divorced or unwed single mothers; Fu and Wang, 2019). Chinese single mothers who perceive stronger subjective cultural norms against single motherhood may subsequently experience more uncertainty discrepancies related to their single motherhood. Moreover, violation of commonly held cultural norms is associated with social sanctions including reduced opportunities for social interaction, cut-off from personal networks, and withholding of needed resources such as information and support (Mollborn, 2010; Lumino et al., 2016). Hence, we expect that subjective cultural norms against single motherhood serve a source of uncertainty discrepancy for Chinese single mothers and propose that:

**H6:** Stronger cultural norm against single motherhood is associated with more uncertainty discrepancy about how to be a good single mother.

Violation of cultural norms also can lead to negative emotions. That is, people who deviate from cultural norms may experience feelings of anger, shame, anxiety, and guilt, which, in some cases, serve to regulate their future behavior and increase their conformity to a particular social norm (Kam and Bond, 2009; Mollborn, 2010). Therefore, we propose that:

**H7:** Stronger subjective cultural norm against single motherhood is associated with more negative emotional responses.

### Perceived Stigma

Compared to subjective cultural norms which may influence uncertainty discrepancy and negative emotions, perceived stigma may work to inhibit individuals’ information seeking and support seeking behaviors. Goffman (1963) defined stigma as a physical or moral discrediting attribute that reduces the individual from a whole person to a tainted, discounted one because of their deviance from society’s conception of normality. Stigma is contextual in nature, embedded in the norms and values of each distinct culture (Link and Phelan, 2001). In the context of Chinese single motherhood, stigma originates from the deeply rooted and widely held cultural values that uphold the moral logics of self-discipline for social and family order (Jeffreys, 2006).

**FIGURE 1:**
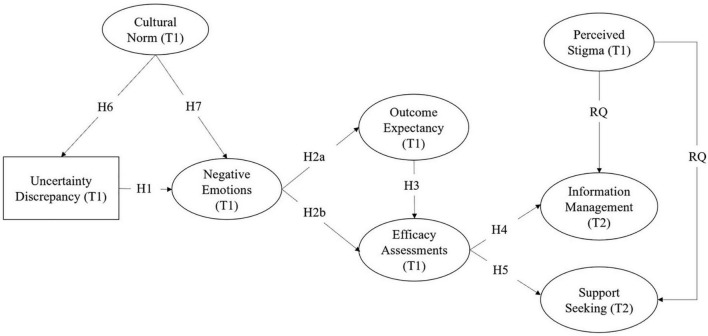
The hypothesized model.

Perceived stigma of a target issue (e.g., single motherhood) and patterns of stigmatization within individuals’ cultural communities may lead to avoidance (Tang and Bie, 2016). Specifically, people who feel stigmatized may enact techniques of information control, such as concealing stigmatized identities and selectively revealing their information needs in order to fit in Chang and Bazarova (2016). Individuals living with stigmatized identity are cautious about whom they disclose their status to and whom they seek information and support from due to fear of rejection, communication difficulties, and a desire to protect the other person (Derlega et al., 2004). Moreover, self-disclosure to seek desired information and support may result in the loss of control over the information and greater stress, a threat that the stigmatized individual needs to evaluate (Brashers et al., 2004; Peterson, 2010). In these cases, single mothers who experience stigma because of the negative connotations attached to divorced and unwed childbirth in Chinese society (Zhang et al., 2014; Fu and Wang, 2019) may turn to online communities to seek what they need. Hence, we explore the effects of perceived stigma on Chinese single mothers’ information management and support seeking decisions within interpersonal (i.e., family and friends) and online contexts and propose the following research question (see Figure 1 for the proposed model):

**RQ:** How do perceived stigma impact Chinese single mothers’ information management and support seeking about being a good single mother offline and online?

## Materials and Methods

### Participants and Procedures

Participants were recruited to participate in a two-wave, longitudinal study through WeChat, the largest social networking platform in China. WeChat accounts for over 1.13 billion monthly active users, with 8 out of 10 Chinese smartphone owners on WeChat (Chen et al., 2018; Statista Research Department, 2019). Anticipating difficulties in reaching single mothers due to the sensitivity of the topic, the authors partnered with One Mom,^2^ a non-profit organization centered on single mother empowerment. Established in 2015, the organization now maintains more than 20 groups on WeChat with over 10,000 members. Our collaboration with the organization also helped validate the identity of the participants as single mothers.

Recruitment messages were posted to single mothers’ WeChat groups. Single mothers interested in participating in the study provided informed consent and completed Time 1 surveys that included measures of validated TMIM constructs (i.e., uncertainty discrepancy, negative emotions, outcome, and efficacy assessments), subjective cultural norm, and perceived stigma. All measures were translated into Chinese and back-translated into English by the first and second authors fluent in both Chinese and English. The authors discussed the appropriateness of the translation and pilot tested the survey with a small Chinese sample (*n* = 14) prior to data collection for the primary study. Individuals who participated at Time 1 were invited to take a follow-up survey 4 weeks later and reported their actual information seeking, avoidance, and direct/indirect support seeking behaviors in the past 4 weeks. After removing data that failed quality check (i.e., minimum time spent on the survey, attention check questions, straightlining responses), the final dataset included responses from 226 Chinese single mothers at both waves.

Most of the participants (65.6%) were between the ages of 30–39 and 20.7% were between the ages of 40–49, 12.4% below 29 and 1.3% above 50. Participants reported their marriage status as 13% unmarried, 81% divorced and 6% widowed. Majority of the participants (81.4%) have a monthly income of 3,000 CNY or above. Their duration of single motherhood ranged from 2 months to 16 years, with an average year of single motherhood was 3.41 years (*SD* = 2.66).

### Measures

For all scales, *M*, *SD*, and reliability were calculated and reported. Confirmatory factor analyses (CFAs) were conducted using AMOS 26 for scales with four or more items. Model fit was considered acceptable if: (a) CFI value was above 0.90 (Hu and Bentler, 1999), (b) root mean square error of approximation (RMSEA) was below 0.08, and (c) relative chi-square (χ^2^/df) of 5 or less (Kline, 2016). All measures used in this study are presented in Supplementary Appendix A.

### Uncertainty Discrepancy

Uncertainty discrepancy was calculated by taking the difference between how much respondents already knew and how much they desired to know about single motherhood. Consistent with prior research (e.g., Afifi and Afifi, 2009), an index was created by subtracting participants’ response to the question “How much information do you have about being a single mother?” from their answer to the question “How much information do you need to have about being a single mother?” on a 7-point scale (1 = *nothing*; 7 = *everything*). Participants’ uncertainty discrepancy scores ranged from −4 to 6 (*M* = 1.82, *SD* = 2.19). Most participants (70.4%) had positive scores, indicating higher-than-desired uncertainties in the context of single motherhood.

### Negative Emotions

Consistent with Fowler and Afifi (2011), uncertainty discrepancy-related negative emotions were measured by asking participants to rate the extent to which they experienced 12 possible negative emotional responses when thinking about the difference between their current and desired levels of knowledge about being a single mother (i.e., anxious, worried, sad, guilty, nervous, scared, disappointed, distressed, frustrated, upset, irritable, and angry). Participants rated their emotional responses to uncertainty discrepancy perceptions on a 5-point scale (1 = *Not at all* to 5 = *Extremely*). Higher scores indicated greater levels of negative emotions (α = 0.95, *M* = 2.55, *SD* = 0.91).

### Outcome Expectancy

Two items were adapted from Afifi and Weiner (2006) and used to assess the extent to which respondents felt that seeking information would produce positive outcomes (e.g., “Asking others about how to be a good single mother would produce ___”). The items were measured on 5-point Likert-type scales (1 = *extremely negative results* to 5 = *extremely positive results*), with higher scores indicating more positive expectations (α = 0.65, *M* = 3.20, *SD* = 0.67).

### Efficacy

Three types of efficacy assessments were assessed using validated measures (Afifi and Weiner, 2006; Afifi et al., 2006). Specifically, communication efficacy was measured with three items that asked about the extent to which respondents felt confident in their ability to initiate conversations with their family, friends, and other people (e.g., “I feel like I have the ability to approach my families to ask about advice to be a single mother;” α = 0.71, *M* = 3.13, *SD* = 0.88). Coping efficacy was measured with five items (e.g., “I feel I can manage discovering more information about being a single mother;” α = 0.94, *M* = 3.79, *SD* = 0.86). Target efficacy was defined as respondents’ perceptions of the target’s willingness and ability to provide information about how to be a good single mother. One item was used to measure the extent to which respondents believed that the target was able to provide information about being a single mother (i.e., “I feel that my families and friends could provide me with information about being a single mother”). Two items were used to measure the degree to which they perceived the target would respond honestly to questions about single motherhood (e.g., “I feel that my families and friends would be completely honest about their advice related to being a single mother”). Together, the three items formed a reliable index (Cronbach’s α = 0.91, *M* = 2.73, *SD* = 0.97). All items were measured on 5-point Likert-scales, with higher scores indicating greater efficacy levels. CFA results suggested that a second-order factor with efficacy as the higher-order latent factor and communication, coping, and target efficacy as the first-order factors fit the data well, χ^2^/df = 2.185, CFI = 0.965, RMSEA = 0.073.

### Information Seeking and Avoidance

Findings from the pilot study indicated that most respondents sought information from their friends, family, and online communities. Therefore, we measured information seeking and avoidance behaviors from these three sources at Time 2. Participants were asked to rate the extent to which they had engaged in information seeking and information avoidance since the first survey (i.e., in the past 4 weeks). Three items adapted from previous research (e.g., Dillow and Labelle, 2014) were used to assess information seeking offline (e.g., “In the past 4 weeks, I sought advice from my friends/family about how to manage being a single mother”). Both measures of information seeking from friends (α = 0.92, *M* = 2.40, *SD* = 0.90) and from family (α = 0.89, *M* = 1.93, *SD* = 0.83) showed high reliability.

Four items adapted from Tian et al. (2016) were used to measure information avoidance offline (e.g., “In the past 4 weeks, I avoided discussing topics related to being a single mother with my friends/family”). Both measures of information avoidance from friends (α = 0.84, *M* = 2.72, *SD* = 1.02) and information avoidance from family (α = 0.80, *M* = 2.96, *SD* = 1.05) were reliable and unidimensional (χ^2^/df = 2.290, CFI = 0.996, RMSEA = 0.076 for avoidance from family; χ^2^/df = 0.101, CFI = 1.000, RMSEA = 0.000 for avoidance from friends).

Two items adapted from Dillow and Labelle (2014) were used to assess online information seeking since Time 1 survey (e.g., “In the past 4 weeks, I sought advice from online sources about how to manage being a single mother;” *r* = 0.882, *M* = 3.04, *SD* = 1.02). Two items assessed online information avoidance (e.g., “In the past 4 weeks, I avoided discussing topics related to being a single mother online;” *r* = 0.717, *M* = 1.77, *SD* = 0.82). The items were measured on 5-point Likert-type scales with higher scores indicated greater levels of information seeking and avoidance.

### Support Seeking

Items used to measure direct and indirect support seeking were adapted from Derlega et al. (2004). Specifically, four items assessed direct support seeking since Time 1 (e.g., “I asked my friends/family how I can best handle being a single mother in the past 4 weeks”). Both measures of direct support seeking from friends (α = 0.93, *M* = 2.47, *SD* = 0.91) and direct support seeking from family (α = 0.89, *M* = 2.01, *SD* = 0.82) produced high reliability. CFAs also yielded good model fit for both family (χ^2^/df = 2.215, CFI = 0.995, RMSEA = 0.073) and friends (χ^2^/df = 0.342, CFI = 1.000, RMSEA = 0.000) after correlating one pair of error terms.

Four items were used to assess indirect support seeking (e.g., “In the past 4 weeks, I fidgeted a lot in front of my friends/family when I had issues with being a single mother”). The items formed reliable and unidimensional scales for both friends (α = 0.91, *M* = 1.85, *SD* = 0.76) and family (α = 0.87, *M* = 2.01, *SD* = 0.82). CFAs were conducted to test the appropriateness of the four-item measure and yielded good model fit for both family (χ^2^/df = 0.936, CFI = 1.000, RMSEA = 0.000) and friends (χ^2^/df = 0.715, CFI = 1.000, RMSEA = 0.000) after correlating one pair of error terms.

### Subjective Cultural Norm

Three items adapted from Fishbein (1975) and Azjen and Fishbein (1980) were used to assess subjective cultural norm (e.g., “Most people who are important to me think I should not be a single mother”). The items formed a reliable index (α = 0.94, *M* = 2.36, *SD* = 1.13).

### Perceived Stigma

An 11-item measure was adapted from Hong et al. (2010) and used to assess participants’ perceived stigma from the community (e.g., “I feel that if I disclosed being a single mother to people in my community, they would not talk to me anymore”). The items formed a reliable (α = 0.96, *M* = 2.45, *SD* = 0.89) and unidimensional scale, χ^2^/df = 1.930, CFI = 0.991, RMSEA = 0.064. Five items were used to measure participants’ perceived stigma from the family (e.g., “I feel that if I disclosed being a single mother to my family, they would not talk to me anymore”). The items also formed a reliable (α = 0.92, *M* = 2.14, *SD* = 1.03) and unidimensional scale (χ^2^/df = 1.874, CFI = 0.996, RMSEA = 0.062).

### Issue Importance

Issue importance is the scope condition for the TMIM and was measured with the item “How important is it to know a lot about being a single mother?” (1 = *not at all* to 5 = *extremely important*; *M* = 3.90, *SD* = 0.811), suggesting that the scope condition was met. Issue importance was included as a covariate in all analyses.

## Results

H1 predicted that uncertainty discrepancy about single motherhood would be positively associated with negative emotions. H2 proposed that negative emotions resulting from uncertainty discrepancy would be negatively associated with (a) outcome expectancies and (b) efficacy assessments. H3 predicted that outcome expectancies would be positively associated with efficacy assessments. H4 suggested that efficacy assessments would be (a) positively associated with information seeking and (b) negatively associated with avoidance. H5 proposed that efficacy assessments would be (a) positively associated with direct support seeking and (b) negatively associated with indirect support seeking. H6 predicted that stronger cultural norm against single motherhood would be associated with more uncertainty discrepancy. H7 proposed that cultural norm would be positively associated with negative emotions. Finally, the RQ asked how perceived stigma would impact Chinese single mothers’ information management and support seeking behaviors.

To test the hypotheses and address the RQ, structural equation modeling (SEM) was tested using AMOS 26. Specifically, uncertainty discrepancy was entered in the model as an observed variable. All other constructs were modeled as latent variables with observed indicators. Given the size of the models, item parceling was used (Matsunaga, 2008), with two to three parcels per latent variable. Separate models were tested using maximum likelihood estimation for each relational context (i.e., family, friends, and online; see Figure 1 for proposed model). Age, monthly income, education, length of single motherhood (in years), marriage status, and issue importance were included as covariates and were kept if they had a significant association with any study variables. Table 1 presents bivariate correlations among study variables within the friendship and family contexts. Table 2 presents bivariate correlations among study variables in online community context.

**TABLE 1:** Bivariate correlation of study variables in friendship and family contexts.

Variables	1	2	3	4	5	6	7	8	9	10	11	12
1. UD	-	0.39***	−0.17*	−0.43***	−0.18**	−0.21**	0.27**	0.25**	–0.01	0.09	0.26***	–0.05
2. Emotions	0.39***	_	−0.36***	−0.41***	−0.50***	–0.09	0.43***	0.49***	0.04	0.22***	0.32***	–0.07
3. OE	−0.17*	−0.36***	_	0.38***	0.35***	0.27***	−0.18**	−0.19**	0.06	–0.09	−0.21***	0.02
4. CommEff	−0.43***	−0.41***	0.38***	_	0.50***	0.57***	−0.23***	−0.25***	0.13*	–0.11	−0.37***	0.19**
5. CopEff	−0.18***	−0.50***	0.35***	0.50***	_	0.29***	−0.40***	−0.27***	–0.04	−0.31***	−0.30***	0.03
6. TarEff	−0.21***	–0.09	0.27***	0.57***	0.29***	_	–0.02	–0.12	0.23***	0.01	−0.28***	0.24**
7. Stigma	0.21**	0.45***	–0.09	−0.14*	−0.28***	–0.02	_	0.42***	0.02	0.16*	0.28***	0.01
8. Norm	0.25***	0.49***	−0.19**	−0.25***	−0.27***	–0.12	0.59***	_	–0.04	0.08	0.20**	–0.11
9. DSS	0.06	0.04	0.00	0.05	–0.06	0.33***	0.01	–0.05	_	0.61***	−0.48***	0.78***
10. ISS	0.30***	0.24***	−0.16*	−0.18**	−0.34***	–0.04	0.10	0.08	0.31***	_	−0.17*	0.52***
11. Avoidance	0.10	0.30***	–0.12	−0.17**	−0.17*	−0.20**	0.26***	0.32***	−0.37***	0.04	_	−0.46***
12. InfoSeek	0.06	0.05	0.10	0.12	–0.04	0.33***	–0.00	–0.08	0.77***	0.30***	−0.41***	_

*p < 0.05. **p < 0.01. ***p < 0.001.

*UD, uncertainty discrepancy. Emotions, negative emotions. OE, outcome expectancy. CommEff, communication efficacy. CopEff, coping efficacy. TarEff, target efficacy.*

*Norm, subjective cultural norm. DSS, direct support seeking. ISS, indirect support seeking. InfoSeek, information seeking.*

*Upper diagonal shows bivariate correlations of study variables within friendship contexts. Lower diagonal shows bivariate correlations within family contexts.*

**TABLE 2:** Bivariate correlation of study variables in online community context.

Variables	1	2	3	4	5	6	7	8	9	10
1. UD	_									
2. Emotions	0.39***	_								
3. OE	−0.17*	−0.36***	_							
4. CommEff	−0.43***	−0.41***	0.38***	_						
5. CopEff	−0.18***	−0.50***	0.35***	0.50***	_					
6. TarEff	−0.21***	–0.09	0.27***	0.57***	0.29***	_				
7. Stigma	0.27**	0.43***	−0.18**	−0.14*	−0.40***	–0.02	_			
8. Norm	0.25***	0.49***	−0.19**	−0.25***	−0.27***	–0.12	0.42***	_		
9. Avoidance	0.18**	0.28***	−0.16*	−0.17**	−0.27***	–0.08	0.21***	0.16*	_	
10. InfoSeek	0.23***	0.31***	0.10	−0.22***	−0.21**	0.13*	–0.23	0.13	0.05	_

*p < 0.05. **p < 0.01. ***p < 0.001.

*UD, uncertainty discrepancy. Emotions, negative emotions. OE, outcome expectancy. CommEff, communication efficacy. CopEff, coping efficacy. TarEff, target efficacy.*

*Norm, subjective cultural norm. DSS, direct support seeking. ISS, indirect support seeking. InfoSeek, information seeking.*

## Model Testing

All three models showed acceptable model fit (family: χ^2^/*df* = 1.894, CFI = 0.908, RMSEA = 0.063; friends: χ^2^/*df* = 1.969, CFI = 0.913, RMSEA = 0.066; online context: χ^2^/*df* = 2.018, CFI = 0.919, RMSEA = 0.067). Results indicated that across the three contexts, uncertainty discrepancy was positively associated with negative emotions (*b* = 0.224, *p* < 0.001). Negative emotions were negatively associated with outcome expectancies (*b* = −0.353, *p* < 0.001) and efficacy assessments (*b* = −0.398, *p* < 0.001). Outcome expectancies were positively related to efficacy assessments (*b* = 0.330, *p* < 0.001). H1-H3 were supported. In addition, results suggested that cultural norm was positively associated with uncertainty discrepancy (*b* = 0.244, *p* < 0.001). Participants who perceived stronger cultural norms against single motherhood reported experiencing more uncertainty discrepancies. H6 was supported. Stronger perceptions of cultural norms against single motherhood also predicted more negative emotions (*b* = 0.458, *p* < 0.001), supporting H7.

**FIGURE 2:**
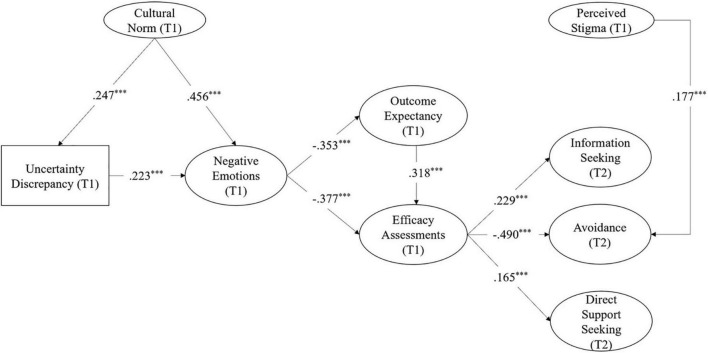
Final model of information management and support-seeking behaviors from friends. Standardized path coefficients are shown in the figure. Covariates included in the model are not shown in the figure. ***p < 0.001.

In the model within friendship context (see Figure 2 for the final model), higher efficacy assessments at Time 1 led to more information seeking (*b* = 0.229, *p* = 0.004), more direct support seeking (*b* = 0.165, *p* = 0.033), and less avoidance (*b* = −0.490, *p* < 0.001) from friends at Time 2, supporting H4a, H4b, and H5a. However, efficacy assessments were not significantly associated with indirect support seeking from friends (*b* = −0.145, *p* = 0.008). H5b were not supported in the model with friends. Regarding RQ, results indicated that those who reported higher levels of perceived stigma from community members at Time 1 engaged in more avoidance behaviors with friends at Time 2 (*b* = 0.177, *p* = 0.006). Perceived stigma was not associated with information seeking (*b* = 0.065, *p* = 1.526) or support seeking from friends (*b* = 0.072, *p* = 1.260).

In the model within family context, higher efficacy assessments at Time 1 led to less indirect support seeking (*b* = −0.313, *p* < 0.001) and less avoidance (*b* = −0.261, *p* = 0.002) from family members at Time 2, supporting H4b and H5b. However, efficacy assessments were not significantly associated with information seeking (*b* = 0.13, *p* = 0.118) or direct support seeking from family (*b* = 0.088, *p* = 0.083); H4a and H5a were not supported. Regarding RQ, results indicated that those who reported higher levels of perceived stigma from family members at Time 1 engaged in more avoidance behaviors at Time 2 (*b* = 0.268, *p* < 0.001).

Last, in online context, efficacy assessments at Time 1 only predicted less avoidance behaviors at Time 2 (*b* = −0.314, *p* < 0.001), but not information seeking (*b* = −0.206, *p* = 0.136). H4b was supported, whereas H4a was not supported. For RQ, perceived stigma from community at Time 1 led to more online information seeking at Time 2 (*b* = 0.235, *p* < 0.001). We discuss theoretical and practical implications of these findings below.

## Discussion

This longitudinal study drew from the TMIM to examine cultural factors, including subjective cultural norms and perceived sigma, associated with Chinese single mothers’ information seeking and support seeking behaviors. Analyses using two-wave data suggest that subjective cultural norms against single motherhood served as sources of uncertainty discrepancies and negative emotions for Chinese single mothers, whereas perceived stigma directly impacted communication behaviors. Consistent with previous TMIM literature, Chinese single mothers’ uncertainty discrepancies about how to be a good single mother accounted for their negative emotions, which were negatively associated with outcome expectancies and efficacy assessments. The effects of efficacy on information seeking, avoidance, and support seeking strategies differed depending on relational contexts (i.e., friends vs. family vs. online communities). Theoretical and practical implications of these findings are discussed below.

### Theoretical Implications

The TMIM has been primarily tested using samples in Western countries (see Kuang and Wilson, 2021 for a meta-analysis). The degree to which the TMIM is culturally relevant in non-Western cultural contexts has received limited attention (see Chang, 2014 for an exception). This study was the first to apply the TMIM to the context of single motherhood in China, extending theoretical understanding about the applicability of TMIM in non-English speaking contexts. The TMIM emphasizes individuals’ active role in information management and serves as a suitable theoretical framework for the analyses of Chinese single mothers’ search for information and support in response to their perceived uncertainty discrepancies about single motherhood. Two-wave data showed general support for the paths proposed by the TMIM. Specifically, the associations among uncertainty discrepancy, negative emotions, outcome expectancies, and efficacy assessments were consistent with results from other TMIM studies, as was the negative association between efficacy assessments at Time 1 and avoidance at Time 2 across the relational contexts of friendship, family, and online communities (e.g., Afifi and Afifi, 2009).

However, the relationships between efficacy and information seeking and support seeking showed heterogeneity across different relational contexts. Efficacy assessments only predicted Chinese single mothers’ search of information from friends, but from not family members or online communities. One possible explanation is that participants may see more value in acquiring information from their friends rather than their families, as single motherhood may be considered a shame to their families by most single mothers (Zhang et al., 2014). In this case, even with high levels of efficacy assessments, single mothers may still refrain from seeking information from family members. This also suggests that information seeking and avoidance are not binary (Babrow, 2001); in this study, lower efficacy predicted more avoidance, but higher efficacy did not necessarily lead to more information seeking. Future research should continue to examine factors that may moderate the association between efficacy assessments and information seeking.

This study also extends the TMIM by identifying possible sources of uncertainty discrepancy perceptions. The TMIM delineates information management processes as a process of individuals-level factors (e.g., uncertainty discrepancy, emotions, and efficacy assessments). However, these processes need to be situated within broader social and cultural contexts. In this study, we examined the roles of subjective social norms and perceived stigma in the information management and support seeking processes. Specifically, in the Chinese cultural context, single motherhood typically is seen as a violation of cultural norm; therefore, subjective cultural norm contributed to uncertainty discrepancies related to single motherhood and associated negative emotions. In comparison, perceptions of stigma directly impacted communication behaviors. For example, perceived stigma led to more avoidance in interpersonal contexts and more information seeking from online sources. These findings contribute to a more sophisticated understanding of information management processes situated within unique cultural (e.g., Chinese) and relational (e.g., friends, family) contexts.

Overall, the findings suggest an inhibition effect of perceived stigma on individuals’ information seeking from interpersonal channels, which drove single mothers to seek needed information from online platforms. These findings highlight the Internet as a valuable resource for individuals to access information on socially stigmatized topics. Computer-mediated information and support seeking may empower the stigmatized and marginalized group and facilitate enhancement of overall wellbeing (Van Uden-Kraan et al., 2009; Oh and Lee, 2012). Women’s online information seeking and support seeking behaviors are of particular scholarly and practical interest. The growth of the Internet and social networking sites in China shows their potential to disrupt established patterns of gender expectations and provide easy access to information and social support (Giordano et al., 2007; Humphreys and Vered, 2014). Future research should continue to understand information management processes *via* interpersonal as well as mediated channels (Afifi, 2015), examine online information seeking for other disadvantaged and minority groups in stigmatized contexts, and explore how to best motivate these individuals to seek useful information.

### Practical Implications

The findings have practical implications. Previous research suggests that the experience of divorced and single motherhood is characterized by ambivalence and uncertainty as well as negative emotions and stress, indicating a need for information and support (Hung et al., 2004; Hertog, 2009). Subjective cultural norms against single motherhood exacerbate uncertainty discrepancies and negative emotions. To alleviate the negative emotions and stress that single mothers experience and facilitate effective uncertainty management, it may be necessary to design and implement targeted efforts to transform the widely held cultural norms and normalize single motherhood.

In addition, lack of information and support seeking, especially from families, shows that Chinese single mothers encounter substantial barriers to openly discuss topics about how to be a good single mother and acquire social support. To assist Chinese single mothers’ information and support seeking, communication interventions should focus on creating a safe and inclusive environment in which single mothers feel less stigmatized. These interventions may involve training for family and friends on critical evaluation of and resistance to stigma perceptions surrounding single mothers. With lower levels of perceived stigma, women may feel more open and comfortable to engage in dialogue with their families and friends and subsequently seek the information and support needed. Large-scale communication campaigns that aim at breaking social taboos against talking about single motherhood in the Chinese society can also encourage information and support seeking as well as provision for single mothers in their social networks.

### Limitations

The findings of the study need to be considered in light of several limitations. First, the study relied on self-report data rather than observational or objective data regarding participants’ information management and support seeking behaviors. Future research should consider additional forms of data collection, such as daily diary methods to document and reflect on the amount of time single mothers spend in online support communities or recording the number of clicks in actual information seeking. Second, although we adopted a longitudinal design in this study, there were still a number of scales included within the two surveys (especially in Time 1 survey). Therefore, the results should be interpreted with considerations of common method bias. Also, the size of the sample was relatively small in this study. Due to the sensitive nature of single motherhood in China, it was challenging to obtain a larger sample. However, by collaborating with a local NGO, we were able to collect responses from rural and urban areas across 24 provinces in China which resulted in a fairly geographically representative sample. Future research can continue to explore strategies to collect data from hard-to-reach populations in specific cultural contexts.

In conclusion, this study examined Chinese single mothers’ information and support seeking from families, friends, and online sources. Findings supported the utility of the TMIM and contributed to better understandings of perceived stigma and cultural norms in individuals’ uncertainty management processes. Our study also suggested the need for more research on information management and support seeking processes regarding taboo topics and consideration of cultural factors that may influence these processes.

## Data Availability Statement

The raw data supporting the conclusions of this article will be made available by the authors, without undue reservation.

## Ethics Statement

The studies involving human participants were reviewed and approved by the Bloomsburg University—IRB. The patients/participants provided their written informed consent to participate in this study.

## Author Contributions

KK: conceptualization (lead), investigation (equal), methodology (lead), formal analysis (lead), writing–original draft (equal), writing–review, and editing (equal). XZ: conceptualization (equal), investigation (lead), methodology (equal), formal analysis (equal), writing–original draft (lead), writing–review, and editing (equal). IB and TH: conceptualization (equal), investigation (equal), methodology (equal), formal analysis (equal), writing–original draft (equal), writing–review, and editing (lead). All authors contributed to the article and approved the submitted version.

## Conflict of Interest

The authors declare that the research was conducted in the absence of any commercial or financial relationships that could be construed as a potential conflict of interest.

## Publisher’s Note

All claims expressed in this article are solely those of the authors and do not necessarily represent those of their affiliated organizations, or those of the publisher, the editors and the reviewers. Any product that may be evaluated in this article, or claim that may be made by its manufacturer, is not guaranteed or endorsed by the publisher.

## Footnotes

1 For instance, according to the Administrative measures of Beijing Municipality on the collection of social maintenance fees (People’s Government of Beijing Municipality, 2021), for every child born out of wedlock, a penalty called “the social fostering fee” is charged to the child’s father or mother, with the amount equating to a half of the annual per capita income.

2 “One Mom” is a non-profit organization headquartered in Beijing, China. It provides various educational programs to empower and encourage single mothers in China.
