# Supplementary material for: From Offline to Online: Understanding Chinese Single Mothers’ Uncertainty Management in Interpersonal and Online Contexts
Source: Front Psychol. 2022 May 6;13:845760. doi: 10.3389/fpsyg.2022.845760 (PMC9120956; doi:10.3389/fpsyg.2022.845760)
Supplement: Supplementary file 1 [file Table_1.docx]

**Appendix A**

**Sample Items Used in the Study**

**Issue Importance** (1 = *not at all* to 5 = *extremely important*)

- How important is it to know a lot about being a single mother?

**Uncertainty Discrepancy** (1 = *nothing* to 7 = *everything*)

1. How much information do you have about being a single mother?
2. How much information do you need to have about being a single mother?

**Negative Emotions** (1 = *not at all* to 5 = *extremely*)

- To what extent do you experience the following feelings and emotions when thinking about how much/little you know about being a single mother?

Anxious

Worried

Sad

Guilty

Nervous

Scared

Disappointed

Distressed

Frustrated

Upset

Irritable

Angry

**Outcome Expectancy** (1 = *extremely negative results* to 5 = *extremely positive*)

1. Asking others about how to be a (good) single mother would produce _______ results.
2. A search for information about how to be a single mother would produce _________ information.

**Communication Efficacy** (1 = *not at all* to 5 = *always*)

1. I feel like I have the ability to approach my families/friends to ask about advice to be a single mother.
2. I know how to talk to my families/friends about being a single mother.
3. I know how to where to go to find out more about being a (single) mother.

**Coping Efficacy** (1 = *not at all* to 5 = *always*)

1. I feel I can manage discovering more information about being a single mother.
2. I’d be able to fully cope with my family’s/friends’ opinions about being a single mother, even if they are negative.
3. I’d be able to fully cope with my family’s/friends’ opinions about being a single mother, even if they are negative.
4. I’d be able to fully cope with family’s/friends’ opinions about being a single mother, even if they are negative.
5. I’d be able to fully cope with online information about being a single mother, even if they are negative.

**Target Efficacy** (1 = *never* to 5 = *always*)

1. I feel that my families and friends could provide me with information about being a single mother.
2. I feel that my families and friends would be completely honest about their advice related to being a single mother.
3. My families and friends would be forthcoming about sensitive issues that I would need to know about being a single mother.

**Information Seeking Offline** (1 = *not at all* to 5 = *very much*)

1. In the past four weeks, I sought advice from my friends/family about how to manage being a single mother.
2. In the past four weeks, I talked to my friends/family about being a single mother.
3. In the past four weeks, I sought information about being a single mother from my friends/family.

**Information Avoidance Offline** (1 = *not at all* to 5 = *very much*)

1. In the past four weeks, I avoided discussing topics related to being a single mother with my friends/family
2. In the past four weeks, I avoided seeking support from my friends/family related to being a single mother.
3. In the past four weeks, I did not seek any support from friends/family about being a single mother.
4. In the past four weeks, I went out of my way to avoid information about this issue.

**Information Seeking Online** (1 = *not at all* to 5 = *very much*)

1. In the past four weeks, I sought advice from online sources about how to manage being a single mother.
2. In the past four weeks, I sought information about being a single mother online.

**Online Information Avoidance** (1 = *not at all* to 5 = *very much*)

1. In the past four weeks, I avoided discussing topics related to being a single mother online.
2. In the past four weeks, I tried not engage in conversations related to being a single mother online.

**Direct Support Seeking Offline** (1 = *not at all* to 5 = *very much*)

1. I asked my friends/family how I can best handle being a single mother in the past four weeks.
2. I told my friends/family the specific details of my uncertainties.
3. I asked my friends/family for help with issues related to being a single mother.
4. I told my friends/family the exact emotions I am experiencing related to being a single mother.

**Indirect Support Seeking Offline** (1 = *not at all* to 5 = *very much*)

1. In the past four weeks, I fidgeted a lot in front of my friends/family when I had issues with being a single mother.
2. In the past four weeks, I complained to my friends/family about problems related to being a single mother.
3. In the past four weeks, I sighed a lot around my friends/family when I had a problem with being a single mother.
4. In the past four weeks, when I was with my friends/family, I made sounds of irritation and move in an angry fashion when I was upset with being a single mother.

**Subjective Cultural Norm** (1 = *totally disagree* to 5 = *totally agree*)

1. Most people who are important to me think I should not be a single mother.
2. Most people whose opinion I value think I should not be a single mother.
3. Most people who are close to me think I should not be a single mother.

**Perceived Stigma from Community** (1 = *not at all* to 5 = *always*)

1. I feel that if I disclosed being a single mother to people in my community, they would not talk to me anymore.
2. I feel that if I disclosed being a single mother to some people, they would think badly of me.
3. I feel that if I disclosed being a single mother to some people, they would treat me differently
4. I feel that if I disclosed being a single mother to some people, they would be uncomfortable around me
5. I feel that if I disclosed being a single mother to some people, they would look down on me.
6. I feel that if I disclosed being a single mother to some people, they would think less of me.
7. I feel that if I disclosed being a single mother to some people, they would be disgusted by me
8. I feel that if I disclosed being a single mother to some people, they would not want to be friends with me
9. I feel that if I disclosed being a single mother to some people, they would think I was immoral
10. I feel that if I disclosed being a single mother to some people, they would avoid me
11. I feel that if I disclosed being a single mother to some people, they would gossip about me

**Perceived Stigma from Family** (1 = *not at all* to 5 = *always*)

1. I feel that if I disclosed being a single mother to my family, they would not talk to me anymore.
2. I feel that if I disclosed being a single mother to my family, they would think badly of me
3. I feel that if I disclosed being a single mother to my family, they would feel ashamed
4. I feel that if I disclosed being a single mother to my family, they would treat me differently
5. I feel that if I disclosed being a single mother to my family, they would think I was immoral
